# Depression is related to dietary diversity score in women: a cross-sectional study from a developing country

**DOI:** 10.1186/s12991-017-0162-2

**Published:** 2017-11-16

**Authors:** Mina Poorrezaeian, Fereydoun Siassi, Alireza Milajerdi, Mostafa Qorbani, Javad Karimi, Reza Sohrabi-Kabi, Neda Pak, Gity Sotoudeh

**Affiliations:** 1 Department of Community Nutrition, School of Nutritional Sciences and Dietetics, Tehran University of Medical Sciences, Hojatdost Street, Naderi Street, Keshavarz Blv., Tehran, Iran; 2 Non-communicable Diseases Research Center, Alborz University of Medical Sciences, Karaj, Iran; 3 Department of Psychology, School of Literature and Humanity Sciences, Malayer University, Malayer, Iran; 4 Ibn Sina Psychiatric Hospital, Hormozgan University of Medical Sciences, Bandar Abbas, Iran; 5 Shariati Hospital, School of Medicine, Tehran University of Medical Sciences, Tehran, Iran; 6 Children Hospital of Excellence, School of Medicine, Tehran University of Medical Sciences, Tehran, Iran

**Keywords:** Depression, Stress, Dietary diversity score, Women

## Abstract

**Background:**

Substantial evidence provides support for the role of diet in the prevention and control of mental disorders. However, since there is no study regarding the relationship between dietary diversity and stress or depression, we aimed to determine the relationship between the dietary diversity score (DDS) and stress and depression in women.

**Methods:**

This descriptive-analytical cross-sectional study was performed on 360 women aged 20–49 years attending health centers in the south of Tehran. The dietary intake and score of depression, anxiety, and stress were measured using a 24-h dietary recall and the 42-item depression, anxiety, stress scales questionnaire, respectively. The DDS was calculated based on the FAO 2013 guidelines. Data were analyzed using Chi-square, analysis of variance, Spearman correlation coefficient, and multivariable logistic regression tests.

**Results:**

In total, 31.4 and 25.8% of the subjects suffered from depression and stress, respectively. After adjusting for confounders, a one-unit increase in DDS was associated with a 39% reduction in the risk of severe depression. The DDS was not significantly associated with mild or moderate depression, and no significant relationship was observed between the DDS and stress.

**Conclusions:**

The DDS could be inversely associated with depression in women. Since we observed no significant relationship between stress and DDS, further studies are needed in this regard.

## Background

The burden of mental disorders continues to grow with significant impacts on health in all countries of the world [1]. According to the World Health Organization (WHO), unipolar depressive disorders are the third greatest burden of disease globally [2]. Several investigations have revealed that diet, as a modifiable lifestyle factor, could have a major role in the prevention and control of mental disorders. These studies have mostly focused on the relationship between individual nutrients or dietary patterns with the risk of depression and stress [3–5]. However, despite the role of individual nutrients, it is important to note that there are still many unidentified compounds in different foods and potential interactions between the nutrients. Therefore, dietary pattern analysis seems more reasonable instead of evaluation of individual nutrients or foods [6].

Based on United States Department of Agriculture (USDA) guidelines and the Food Guide Pyramid, dietary diversity is one of the characteristics of a healthy diet. Dietary diversity represents the consumption of various food items within and between food groups [7]. Several indices are available for dietary quality assessment, including the Healthy Eating Index (HEI) [8] and dietary diversity score (DDS) [9].

The DDS is a simple, efficient, and low-cost index for dietary quality assessment. In addition, it is easy to answer [10]. For calculating the DDS, the FAO recommends using a single 24-h recall since it is less cumbersome for the respondent. In addition, analysis of data based on a 24-h recall period is reported to be much easier than longer recall periods [11]. The HEI is specified by the Dietary Guidelines for Americans [12]. However, in contrast to the dietary diversity questionnaire, the HEI is reported to be dependent on the ethnicity and culture [13].

In addition to reports of a positive relationship between the DDS and macro- and micro-nutrients intake [14, 15], several investigations have revealed an inverse association between the DDS and chronic diseases, such as metabolic syndrome [16], cardiovascular diseases [17], cancer [18], and hypertension [19]. However, to our knowledge, no previous study has evaluated the relationship between dietary diversity and depression and stress. Women are more vulnerable to depression and stress [20]. Therefore, this study was conducted to determine the relationship between the DDS and depression and stress in women.

## Methods

### Study population

This was a descriptive-analytical cross-sectional study. The detailed methodology of the study has been already reported [21] and is briefly described here. The study was conducted on 360 women aged 20–49 years attending 10 health centers in the south of Tehran, Iran. These centers were randomly selected from 29 health centers in the area. In each selected health center, we defined the number of our subjects in proportion to the total number of patients attending the center. The inclusion criteria were age 20–49 years, a body mass index (BMI) 18.5–34.99 kg/m^2^, and at least primary education. In total, 400 women were invited to participate in the study of whom 40 subjects declined, resulting in a response rate of 90%. The exclusion criteria were pregnancy and lactation, suffering from depression diagnosed by a psychiatrist or using anti-depressive drugs in the past 12 months, history of life stressors or tragic events (such as divorce, financial problems, love failure, death of first degree relatives or friends) in the past 6 months, history of any type of smoking or alcohol consumption, diseases such as diabetes, cardiovascular diseases, hypertension, multiple sclerosis, seizure attacks, or liver, kidney, or thyroid disorders, or regular consumption of any drugs. Since subjects who follow specific diets such as low-calorie diets may have different dietary intakes, we excluded them form the study. Informed written consent form was obtained from all participants. The study protocol and the consent form were approved by the Ethics Committee of Tehran University of Medical Sciences.

### Assessment of depression and stress score

The psychological status of the participants was measured by the Depression, Anxiety, and Stress Scales (DASS-42) questionnaire, which has been validated for use in non-clinical populations and research [22, 23]. This is a 42-item self-report questionnaire, developed by Lovibond and Lovibond [24]. The Cronbach alpha coefficient for the questionnaire was 0.81 for depression, 0.73 for anxiety, and 0.81 for stress in a study of 717 normal subjects in Australia [24]. In another study on 400 high school students in Kermanshah, west of Iran, the coefficient was 0.94 for depression, 0.85 for anxiety, and 0.87 for stress [25]. The DASS-42 contains 14 question items for depression, anxiety, or stress. The answers are divided into four categories as zero, medium–low, medium–high, and highest with a score of 0–3, respectively. Based on the total score, the subjects were divided into five groups of normal (0–9), mild (10–13), moderate (14–20), severe (21–27), and very severe (> 27) depression. These figures for five groups of stress were 0–14, 15–18, 19–25, 26–33, and > 33, respectively. However, due to the limited number of cases in some groups, they were divided into three groups of normal, mild/moderate, and severe.

### Measurement of dietary diversity score

For all participants, a 24-h diet recall questionnaire was completed through face-to-face interviews. The participants’ energy and nutrient intake was calculated by the Nutritionist IV software (N Squared Computing, San Bruno, CA) modified for Iranian foods. In addition, the DDS was calculated based on the FAO (2013) guideline which suggests a questionnaire for the measurement of dietary diversity [11]. This questionnaire contains nine main food groups: (1) cereals, (2) dark green leafy vegetables, (3) vitamin A-rich fruits and vegetables, (4) other fruits and vegetables, (5) organ meat, (6) meat, fish, and seafood, (7) eggs, (8) legumes, nuts, and seeds, and (9) milk and milk products. We defined a scoring system based on the values obtained from the 24-h diet recall questionnaire as described below. The DDS was calculated using a minimum consumption of at least half serving of one food from each mentioned food group. The score of dietary diversity was the total sum of the score of all food groups. A score of 1 was given score for each food group consumed, and the maximum score was 9.

### Covariate assessment

Physical activity was assessed using the short form of the International Physical Activity Questionnaire (IPAQ) during 1 week. The subjects were then divided into three groups of low, moderate, and high physical activity according to the IPAQ criteria [26]. Weight was measured to the nearest 100 g in with minimal clothing and shoes removed. Height was measured in the standing upright position looking straight forward, with heels against the wall without shoes by a stadiometer to the nearest of 0.1 cm. The BMI was calculated as weight (kg) divided by height squared (m^2^). Waist Circumference (WC) was measured midway between the lower rib margin and the iliac crest at the end of a gentle expiration and in the standing position to the nearest of 0.1 cm by a tape. Demographic data, including age, educational level, marital status, monthly household income, history of stressful life events, and dietary supplement intake, were collected.

### Statistical analysis

Statistical analyses were performed by the Statistical Package for Social Sciences (SPSS, version 16; Chicago, IL). Chi square was used to test the relationship between qualitative variables and depression and stress. Analysis of Variance (ANOVA) was applied to assess the relationship between quantitative variables and depression and stress. The correlation between the DDS and energy and nutrient intake was evaluated using the Spearman’s correlation test. Multinomial logistic regression was used to measure the relationship between the DDS and depression and stress, with adjustment for confounding variables.

## Results

In the current study, the mean age of the participants was 32.1 ± 6.3 years, and the prevalence of depression and stress was 31.4 and 25.8%, respectively. The mean DASS score for depression was 27.7 in the severe group as compared to 14.2 in the mild/moderate group and 3.7 in the normal group. These figures were 30.9, 19.2, and 6.7 for stress, respectively.

Based on Tables 1 and 2, depression was significantly associated with age [*F*(2, 357) = 6.16, *p* = 0.002], educational level [*F*(2, 357) = 4.53, *p* = 0.01], energy intake [*F*(2, 357) = 4.93, *p* = 0.008], and monthly household income (*p* = 0.007, *df* = 4), and stress was significantly associated with age [*F*(2, 357) = 2.20, *p* = 0.01], monthly household income (*p* = 0.01, *df* = 4), and physical activity (*p* = 0.01, *df* = 4). There was a significant positive correlation between the DDS and the intake of energy, protein, carbohydrate, fat, B vitamins, fat-soluble vitamins, and minerals including iron, magnesium, zinc, and selenium (*p* < 0.02). In addition, the correlation between the DDS and the percentage of energy obtained from protein, saturated fatty acid (SFA), mono unsaturated fatty acid (MUFA), poly unsaturated fatty acid (PUFA), and docosahexaenoic acid (DHA) was significant (*p* < 0.02). However, we observed no significant correlation between the DDS and the percentage of energy obtained from carbohydrates, fat, and Eicosapentaenoic Acid (EPA) (*p* > 0.05) (Table 3).

**Table 1:** Association of general characteristics of participants with depression status

Characteristics	Depression status			*F*	*df* ^a^	*df* ^b^	*p* value	*p* trend
	Normal (*n* = *247*) M ± SD or *n* (%)	Mild/moderate (*n* = *81*) M ± SD or *n* (%)	Severe (*n* = *32*) M ± SD or *n* (%)					
DASS (score) for depression	3.7 ± 2.7	14.2 ± 3.1	27.7 ± 4.3					
Age (years)	32.8 ± 6.2	30.0 ± 6.6	32.3 ± 5.6	6.1*	2	357	0.002**	0.03
Education (years)	11.6 ± 3.2	10.9 ± 3.2	10.0 ± 3.08	4.5*	2	357	0.01**	0.003
Weight (kg)	67 ± 9.9	67.1 ± 10.9	69.5 ± 8	0.8*	2	357	0.4**	0.3
Height (cm)	158.4 ± 5.4	158.8 ± 4.8	160 ± 5.3	1.1*	2	357	0.3**	0.1
Body mass index (kg/m^2^)	26.7 ± 3.5	26.6 ± 4.1	27.2 ± 3.1	0.3*	2	357	0.7**	0.6
Energy (kcal)	1664.4 ± 692.9	1933.6 ± 734.4	1585.9 ± 823.5	4.9*	2	357	0.008**	0.3
Marital status								
Married	233 (94.3)	72 (88.9)	27 (84.4)		2		0.06***	
Unmarried	14 (5.7)	9 (11.1)	5 (15.6)					
Household income (Toman)								
< 500,000	19 (7.8)	11 (13.9)	7 (21.9)		4		0.007***	
500,000–1,000,000	142 (58.2)	49 (62)	22 (68.8)					
≥ 1,000,000	83 (34)	19 (24.1)	3 (9.4)					
Dietary supplement use								
No^c^	197 (79.8)	64 (79)	23 (71.9)		2		0.6***	
Yes^d^	50 (20.2)	17 (21)	9 (28.1)					
Physical activity								
Low	137 (55.5)	47 (58)	19 (59.4)		4		0.07***	
Moderate	95 (38.5)	28 (34.6)	7 (21.9)					
High	15 (6.1)	6 (7.4)	6 (18.8)					

*DASS* depression, anxiety, stress scales

* *F* for ANOVA test

** *p* value is for ANOVA test, *p* < 0.05 is statistically significant *** *p* value is for *X* ^2^ test, *p* < 0.05 is statistically significant

^a^Between groups *df*

^b^Within groups *df*

^c^Less than three times per week

^d^Three or more times per week

**Table 2:** Association of general characteristics of participants with stress status

Characteristics	Stress status			*F*	*df* ^a^	*df* ^b^	*p* value	*p* trend
	Normal (*n* = *267*) M ± SD or *n* (%)	Mild/moderate (*n* = *70*) M ± SD or *n* (%)	Severe (*n* = *23*) M ± SD or *n* (%)					
DASS (score) for stress	6.7 ± 4.0	19.2 ± 3.0	30.9 ± 4.2					
Age (years)	32.5 ± 6.4	30.9 ± 6.3	31.1 ± 4.1	2.2*	2	357	0.01**	0.6
Education (years)	11.5 ± 3.3	11 ± 2.9	10.2 ± 3.1	2.1*	2	357	0.1**	0.003
Weight (kg)	67.2 ± 9.8	66.9 ± 10.6	69.3 ± 9.9	0.5*	2	357	0.6**	0.3
Height (cm)	158.5 ± 5.3	158.9 ± 5.2	160.4 ± 4.7	1.5*	2	357	0.2**	0.1
Body mass index (kg/m^2^)	26.8 ± 3.6	26.5 ± 3.9	26.9 ± 3.7	0.2*	2	357	0.8**	0.6
Energy (kcal)	1724.1 ± 702.8	1734.3 ± 701.3	1597.7 ± 991.3	0.3*	2	357	0.7**	0.6
Marital status								
Married	249 (93.3)	61 (87.1)	22 (95.7)		2		0.2***	
Unmarried	18 (6.7)	9 (12.9)	1 (4.3)					
Household income (Toman)								
< 500,000	24 (9.2)	10 (14.3)	3 (13)		4		0.01***	
500,000–1,000,000	152 (58)	41 (58.6)	20 (87)					
≥ 1,000,000	86 (32.9)	19 (27.1)	0 (0)					
Dietary supplement use								
No^c^	214 (80.1)	54 (77.1)	16 (69.6)		2		0.4***	
Yes^d^	53 (19.9)	16 (22.9)	7 (30.4)					
Physical activity								
Low	148 (55.4)	41 (58.6)	14 (60.9)		4		0.01***	
Moderate	105 (39.3)	21 (30)	4 (17.4)					
High	14 (5.2)	8 (11.4)	5 (21.7)					

*DASS* depression, anxiety, stress scales

* *F* for ANOVA test

** *p* value is for ANOVA test, *p* < 0.05 is statistically significant

*** *p* value is for *X* ^2^ test, *p* < 0.05 is statistically significant

^a^Between groups *df*

^b^Within groups *df*

^c^Less than three times per week

^d^Three or more times per week

**Table 3:** Spearman’s correlation between daily nutrients, energy intake, and dietary diversity score

Dietary diversity score					
Nutrients	r*	*p* value**	Nutrients	r*	*p* value**
Energy (kcal)	0.2	< 0.001	Vitamin D (µg)	0.2	< 0.001
Protein (g)	0.4	< 0.001	Vitamin K (µg)	0.3	< 0.001
Carbohydrate (g)	0.2	< 0.001	Vitamin C (mg)	0.2	< 0.001
Fat (g)	0.2	< 0.001	Folate (µg)	0.4	< 0.001
Protein (% energy)	0.28	< 0.001	Thiamin (mg)	0.3	< 0.001
Carbohydrate (% energy)	− 0.1	0.05	Riboflavin (mg)	0.4	< 0.001
Fat (% energy)	0.03	0.52	Niacin (mg)	0.3	< 0.001
SFA (g)	0.2	< 0.001	Pyridoxine (mg)	0.3	< 0.001
MUFA (g)	0.2	0.001	Cobalamin (µg)	0.3	< 0.001
PUFA (g)	0.1	0.006	Iron (mg)	0.3	< 0.001
DHA (g)	0.1	0.004	Zinc (mg)	0.4	< 0.001
EPA (g)	− 0.5	0.3	Magnesium (mg)	0.3	< 0.001
Vitamin A (RE)	0.4	< 0.001	Selenium (mg)	0.2	< 0.001
Vitamin E (mg)	0.1	0.01			

*SFA* saturated fatty acid, *MUFA* monounsaturated fatty acid, *PUFA* poly unsaturated fatty acid, *DHA* docosahexaenoic acid, *EPA* eicosapentaenoic acid

* *r* is Spearman’s correlation coefficient

** *p* value is for Spearman’s correlation test, *p* < 0.05 is statistically significant

Chi-square test revealed that with increase in DDS tertiles, the percentage of the individuals who consumed vitamin A-rich fruits and vegetables, dark green leafy vegetables, chicken eggs, milk and dairy products, legumes, nuts, and seeds increased (*p* < 0.001, *df* = 2). In addition, the percentage of the individuals who consumed meat, fish, and seafood decreased significantly with increase in the DDS tertiles (*p* < 0.001, *df* = 2). However, there was no significant relationship between the DDS and food groups such as cereals and white roots, organ meat, and other fruits and vegetables (*p* > 0.05, *df* = 2) (Table 4).

**Table 4:** Food groups consumption across the tertiles of dietary diversity score in women

Food groups	Dietary diversity score			Total *n* (%)	*df* ^a^	*p* value^b^
	Tertile 1 (score: 1–4) *n* (%)	Tertile 2 (score: 5) *n* (%)	Tertile 3 (score: 6–7) *n* (%)			
Cereals and white roots	170 (47.2)	120 (33.3)	70 (19.4)	360 (100)	2	0.06
Milk and dairy products	103 (36.8)	111 (39.6)	66 (23.6)	280 (100)	2	< 0.001
Vitamin A-rich vegetables and fruits	4 (9.8)	17 (41.5)	20 (48.8)	41 (100)	2	< 0.001
Green leafy vegetables	20 (15.5)	50 (38.8)	59 (45.7)	129 (100)	2	< 0.001
Other vegetables and fruits	153 (45.8)	114 (34.1)	67 (20.1)	334 (100)	2	0.1
Meat, fish and seafood	118 (41.3)	105 (36.7)	63 (22)	286 (100)	2	< 0.001
Organ meat	5 (33.3)	4 (26.7)	6 (40)	15 (100)	2	0.1
Eggs	14 (20.3)	25 (36.2)	30 (43.5)	69 (100)	2	< 0.001
Nut, seeds and legumes	43 (28.3)	54 (35.5)	55 (36.2)	152 (100)	2	< 0.001

The numbers represent subjects with consumption of at least 0.5 serving of each food group

^a^Between groups *df*

^b^ *p* value is for *X* ^2^ test, *p* < 0.05 is statistically significant

The relationship between the DDS and depression and stress is shown in Tables 5 and 6. In model 1, an increase in DDS led to 38% decrease in the odds ratio (OR) of severe depression. The OR was further reduced after adjustment for potential confounders. Regression models revealed no significant relationship between the DDS and mild or moderate depression (Table 5). As shown in Table 6, no relationship was observed between the DDS and stress in different stress groups in multinomial logistic regression models.

**Table 5:** Odds ratios (OR) with 95% CI for the association between depression and dietary diversity score

Dietary diversity score	Depression status OR (95% CI)			*df* ^a^
	Normal (*n* = 247)	Mild/moderate (*n* = 81)	Severe (*n* = 32)	
Model 1	Reference	0.83 (0.65–1.06)*	0.62 (0.43–0.90)^¥^	1
Model 2	Reference	0.90 (0.68–1.19)*	0.60 (0.41–0.89)^¥^	1
Model 3	Reference	0.82 (0.60–1.11)*	0.61 (0.40–0.91)^¥^	1

Model 1: crude model

Model 2: adjusted for age, education, marital status, household income, physical activity, and energy

Model 3: further adjusted for body mass index (BMI) and dietary supplement intake based on model 2

* *p* > 0.05

^¥^ *p* < 0.05

^a^Between groups *df*

**Table 6:** Odds ratios (OR) with 95% CI for the association between stress and dietary diversity score

Dietary diversity score	Stress status OR (95% CI)			*df* ^a^
	Severe (*n* = 23)	Mild/moderate (*n* = 70)	Normal (*n* = 267)	
Model 1	Reference	0.80 (0.68–1.18)*	0.72 (0.48–1.01)*	1
Model 2	Reference	0.90 (0.68–1.18)*	0.72 (0.48–1.01)*	1
Model 3	Reference	0.89 (0.67–1.18)*	0.76 (0.40–1.16)*	1

Model 1: crude model

Model 2: adjusted for age, education, household income and physical activity

Model 3: further adjusted for body mass index, dietary supplement, and energy intake based on model 2

* *p* > 0.05

^a^Between groups *df*

## Discussion

We observed an inverse association between DDS and severe depression, which remained significant after adjustment for confounders. However, there was no significant association between DDS and stress. To our knowledge, there is no report of the relationship between DDS and depression and stress.

Recent studies have mainly focused on the relationship between nutrients and dietary patterns and depression and stress. One study showed an inverse relationship between depression and a healthy dietary pattern with high consumption of vegetables, fruits, nuts, legumes, olive oil, fish, and low consumption of meat and meat products [4]. In another study on Australian women, a traditional dietary pattern (characterized by vegetables, fruit, meat, fish, and whole grains) was associated with lower risk of depression and the Western dietary pattern (characterized by processed or fried foods, refined grains, and sugary products) was associated with a higher risk of depression [27]. In addition, a healthy dietary pattern is inversely related to depression [28, 29]. However, the result of a meta-analysis showed no association between Western dietary pattern and depression [30].

Based on the results of the current study, an increased DDS was associated with a higher percentage of individuals who consumed food groups such as milk and dairy products, vitamin A-rich fruits and vegetables, dark green leafy vegetables, eggs, legumes, nuts, and seeds. On the contrary, the percentage of individuals who consumed meat, fish, and seafood groups decreased with an increase in the DDS tertiles. This finding might reflect that with an increase in the DDS, subjects decrease their meat intake and increase the intake of other healthy food groups such as fruits, vegetables, and dairy products. In addition, since the price of meat and fish is high in Iran, their consumption is low in many households. The results of a randomized clinical trial suggested that restricting meat, fish, and poultry intake improved some domains of short-term mood state in omnivores [31]. These results are consistent with the findings of another cross-sectional study which showed that vegetarians had better mood than omnivores [32]. Therefore, it can be deduced that with an increase in the DDS, the dietary pattern of the individuals could become more similar to the Mediterranean dietary pattern which is characterized by high intake of fruits, vegetables, whole grains, legumes, nuts, and seeds and low consumption of red meat [33]. The protective role of the Mediterranean dietary pattern against depression has been shown in some studies [34]. In addition, milk and dairy products, fruits and vegetables, eggs, legumes, nuts, and seeds have a high content of B vitamins, particularly vitamins B6, B12, and folic acid [35]. In the current study, a positive correlation was observed between the DDS and dietary intake of vitamins B6, B12, and folic acid. Several epidemiological studies have reported the protective effect of these vitamins against depression [36, 37]. For instance, based on a prospective study on American older adults, high intake of vitamins B6 and B12 is associated with lower incidence of depression after 12 years [36]. Moreover, one meta-analysis showed that after adjusting for confounders, low folate intake was significantly associated with the risk of depression [37]. Serotonin and dopamine deficiency are related to the etiology of depression. Vitamin B6-, B12-, and folate-derived coenzymes are involved in serotonin and dopamine synthesis and metabolism. Furthermore, vitamin B6 and B12 act as a cofactor for the conversion of homocysteine to methionine and cysteine, respectively. Insufficient dietary intake of these vitamins could lead to homocysteine accumulation and reduced synthesis of monoamines in the brain, which might play a crucial role in the etiology of depression [38]. We found a positive correlation between the DDS and the consumption of alpha-linolenic (ALA) and DHA. Based on the percentage distribution of food groups in DDS tertiles, a high DDS was associated with lower consumption of meat and fish, and higher intake of dark green leafy vegetables. So it is possible that the intake of fish, as the primary source of omega-3, is very low in women, as only 6.6% of them declared regular fish intake (data not shown). Therefore, the minor intake of dietary omega-3 fatty acids in our subjects could be attributed to the intake of vegetables. ALA is found in vegetables; this fatty acid can be converted to EPA and DHA in human body. However, fish consumption is considered the main source of EPA and DHA [38]. The results of one meta-analysis revealed that EPA and DHA levels were significantly lower in patients with depression, in comparison with healthy individuals [39]. In neurons, DHA is the primary component of plasma membrane phospholipids, and higher concentration of DHA improves serotonin receptor sensitivity in these cells via increasing the plasma membrane fluidity [40].

Regarding the role of oxidative stress in mental disorders, it has been shown that higher sensitivity of the human brain to oxidative stress could be due to high O_2_ consumption, high intake of iron and polyunsaturated fatty acids, and low activity of antioxidant enzymes [41]. Food groups such as fruits and vegetables are rich in vitamins, carotenoids, polyphenols, and other bioactive compounds, so they provide a great amount of antioxidants [42, 43]. In addition, the riboflavin in milk and dairy products, and flavonoids, plant sterols, and tocopherols in nuts mark these foods as the natural sources of antioxidants [35]. In the current study, the DDS was positively correlated with the consumption of antioxidant nutrients, and increased DDS was associated with higher intake of food groups rich in antioxidants, such as vitamin A-rich fruits and vegetables, legumes, nuts, and seeds. Therefore, since several studies have indicated a positive correlation between a higher DDS and nutrient adequacy [15], increased DDS could be associated with increased consumption of nutrients and antioxidants, which in turn could have an important role in reducing the risk of depression. Furthermore, we previously showed that with an increase in the DDS increased the mean level of blood antioxidant markers, including total antioxidant capacity, superoxide dismutase, and glutathione peroxidase [44].

In the present study, women with mild, moderate, or severe depression had a lower DDS compared to healthy individuals; however, no significant relationship was observed between stress and DDS. Some studies have shown a relationship between perceived stress and unhealthy food consumption [45–47]. Sedaqat et al. examined the association of snacking (fresh fruits, fruit juice, nuts and seeds, sweet beverage, salty snacks, sweet snacks, and fast food) with stress and depression in obese and non-obese women. They found that non-obese subjects in higher tertiles of natural fruit juice had higher ORs for stress, which might reflect higher energy intake with stress [45]. In another study, stress was associated with a lower intake of fruits, vegetables, protein, and higher consumption of salty snacks [46]. Oliver et al. reported a higher consumption of snack-type foods and lower intake of healthy foods such as fruits, vegetables, meat, and fish in stressful periods among university students [47]. Since the causal direction between nutrition and stress is not clear in most of the studies, further investigations are needed to determine the contribution of nutrition, particularly dietary diversity, to the incidence and development of stress.

This study has several limitations. First, the 24-h dietary recall is the main limitation for our study which has several limitations including difficulty in recalling consumed foods, their exact serving size, and under or over reporting [48]. In addition, using one 24-h recall period does not indicate an individual’s habitual diet. Second, no cause-and-effect relationship between DDS and stress and depression could be inferred in this cross-sectional study. There is even the possibility that food inadequacy or low dietary diversity could be the consequence of depression, and not its cause. Some studies have shown that depressed individuals seek to self-medicate with high-fat and high-sugar foods [49, 50]. Third, the sample size of this study is small which affects generalization of the results to the general population, since our subjects were 20- to 45-year-old women attending health centers. Despite these limitations, this is the first study to examine the relationship between DDS and stress and depression. The inclusion and exclusion criteria were well defined, and numerous potential confounding factors were controlled. In conclusion, we observed a significant inverse relationship between dietary diversity and severe, but not mild or moderate, depression in women. These results highlight the importance of a diet characterized by a greater variety and suggest important public health implications for depression prevention. Encouraging the consumption of various foods is important in nutritional interventions aimed at preventing depression. Future research exploring the effect of improved dietary diversity on mental health is warranted.

## References

[1] WHO (World Health Organization). Mental disorders. Fact sheet. http://www.who.int/mediacentre/factsheets/fs396/en/. Accessed Apr 2016.

[2] WHO (World Health Organization). Investing in mental health. Department of mental health and substance dependence. http://www.who.int/healthinfo/global-burden-disease/GBD-report-2004update-full.pdf. Accessed 27 Feb 2013.

[3] Bodnar LM, Wisner KL (2005). Nutrition and depression: implications for improving mental health among childbearing-aged women. Biol Psychiatry.

[4] Rashidkhani B, Pourghassem GB, Ranjbar F, Zareiy S, Kargarnovin Z (2013). Dietary patterns and anthropometric indices among Iranian women with major depressive disorder. Psychiatry Res.

[5] Ansari WE, Adetunji H, Oskrochi R (2014). Food and mental health. Cent Eur J Public Health.

[6] Slattery ML (2010). Analysis of dietary patterns in epidemiological research. Appl Physiol Nutr Metab.

[7] Ruel M (2006). Food consumption and nutrition division.

[8] Overview & background of the healthy eating index. 2015. https://epi.grants.cancer.gov/hei/. Accessed Nov 2016.

[9] Ruel MT (2003). Operationalizing dietary diversity: a review of measurement issues and research priorities. J Nutr.

[10] Hoddinott J, Yisehac Y. Dietary diversity as a food security indicator food and nutrition technical assistance project. Washington, DC: Academy for Educational Development.

[11] Kennedy G, Ballard T, Marieclaude D. Guidelines for measuring household and individual dietary diversity. Nutrition and Consumer Protection Division, Food and Agriculture Organization of the United Nations. 2013.

[12] Guenther PM, Kirkpatrick SI, Reedy J, Kerbs-Smith SM, Buckman DW, Dodd KW, Casavale KO, Carroll RJ (2010). The Healthy Eating Index 2010 is a valid and reliable measure of diet quality according to the 2010 dietary guidelines for Americans. J Nutr.

[13] Guenther PM, Reedy J, Krebs-Smith SM, Reeve BB, Basiotis PP. Development and evaluation of the healthy eating index-2005: technical report center for nutrition policy and promotion, US. Department of Agriculture. 2007. http://www.cnpp.usda.gov/HealthyEatingIndex.htm. Accessed Dec 2016.

[14] Foote JA, Murphy SP, Wilkens LR, Basiotis PP, Carlson A (2004). Dietary variety increases the probability of nutrient adequacy among adults. J Nutr.

[15] Arimond M, Wiesmann D, Becquey E, Carriquiry A, Daniels MC, Deitchler M (2010). Simple food group diversity indicators predict micronutrient adequacy of women’s diets in 5 diverse, resource-poor settings. J Nutr.

[16] Azadbakht L, Mirmiran P, Azizi F (2005). Dietary diversity score is favourably associated with the metabolic syndrome in Tehranian adults. Int J Obesity.

[17] McCullough ML, Feskanich D, Stampfer MJ, Giovannucci EL, Rimm EB, Hu FB (2002). Diet quality and major chronic disease risk in men and women: moving toward improved dietary guidance. Am J Clin Nutr.

[18] Fernandez E, Negri E, LaVecchia C, Franceschi S (2000). Diet diversity and colorectal cancer. Prev Med.

[19] Miller WL, Crabtree BF, Evans DK (1992). Exploratory study of the relationship between hypertension and diet diversity among Saba Islanders. Public Health Rep.

[20] Parker G, Brotchie H (2010). Gender differences in depression. Int Rev Psychiatry.

[21] Poorrezaeian M, Siassi F, Qorbani M, Karimi J, Koohdani F, Asayesh H (2015). Association of dietary diversity score with anxiety in woman. J Psychiatry Res.

[22] Brown TA, Chorpita BF, Korotitsch W, Barlow DH (1997). Psychometric properties of the depression anxiety stress scales (DASS) in clinical samples. Behav Res Ther.

[23] Crawford JR, Henry JD (2003). The depression anxiety stress scales (DASS): normative data and latent structure in a large non-clinical sample. Br J Clin Psychol.

[24] Lovibond PF, Lovibond SH (1995). The structure of negative emotional states: comparison of the depression anxiety stress scales (DASS) with the Beck depression and anxiety inventories. Behav Res Ther.

[25] Afzali A, Delavari A, Borjali A, Mirzamani M (2007). Psychometric properties of DASS-42 as assessed in a sample of Kermanshah high school students. J Res Behav Sci.

[26] IPAQ 2005. Guidelines for data processing and analysis of the international physical activity questionnaire (IPAQ)—short and long forms. IPAQ Research Committee. http://www.ipaq.ki.se. Accessed Jan 2017.

[27] Jacka FN, Pasco JA, Mykletun A, Williams LJ, Hodge AM, O’Reilly SL (2010). Association of Western and traditional diets with depression and anxiety in women. Am J Psychiatry.

[28] Khosravi M, Sotoudeh G, Majdzadeh R, Nejati S, Darabi S, Raisi F (2015). Healthy and unhealthy dietary patterns are related to depression: a case–control study. Psychiatry Investig.

[29] Akbaraly TN, Brunner EJ, Ferrie JE, Marmot MG, Kivimaki M, Singh-Manoux A (2009). Dietary pattern and depressive symptoms in middle age. Br J Psychiatry.

[30] Lai JS, Hiles S, Bisquera A, Hure AJ, McEvoy M, Attia J (2014). A systemic review and meta-analysis of dietary patterns and depression in community-dwelling adults. Am J Clin Nutr.

[31] Beezhold BL, Johnston CS (2012). Restriction of meat, fish, and poultry in omnivores improves mood: a pilot randomized controlled trial. J Nutr.

[32] Beezhold BL, Johnston CS, Daigle DR (2010). Vegetarian diets are associated with healthy mood states: a cross-sectional study in seventh day adventist adults. Nutr J.

[33] Munoz MA, Fito M, Marrugat J, Covas MI, Schroder H (2009). Adherence to the Mediterranean diet is associated with better mental and physical health. Br J Nutr.

[34] Sanchez-Villegas A, Delgado-Rodriguez M, Alonso A, Schlatter J, Lahortiga F, Serra Majem L (2009). Association of the Mediterranean dietary pattern with the incidence of depression: the Seguimiento Universidad de Navarra/University of Navarra. Arch Gen Psychiatry.

[35] Mahan LK, Raymond SS. Krauses food and the nutrition care process. 13 ed. 2012.

[36] Skarupski KA, Tangney C, Li H, Ouyang B, Evans DA, Morris MC (2010). Longitudinal association of vitamin B-6, folate, and vitamin B-12 with depressive symptoms among older adults over time. Am J Clin Nutr.

[37] Gilbody S, Lightfoot T, Sheldon T (2007). Is low folate a risk factor for depression? A meta-analysis and exploration of heterogeneity. J Epidemiol Community Health.

[38] Sanchez-Villegas A, Henriquez P, Bes RM, Doreste J (2006). Mediterranean diet and depression. Public Health Nutr.

[39] Lin PY, Su KP (2007). A meta-analytic review of double-blind, placebo-controlled trials of antidepressant efficacy of omega-3 fatty acids. J Clin Psychiatry.

[40] Peet M, Stokes C (2005). Omega-3 fatty acids in the treatment of psychiatric disorders. Drugs.

[41] Kodydkova J, Vavrova L, Zeman M, Jirak R, Macasek J, Stankova B (2009). Antioxidative enzymes and increased oxidative stress in depressive women. Clin Biochem.

[42] Hermsdorff HH, Barbosa KB, Volp AC, Puchau B, Bressan J, Zulet MA (2012). Vitamin C and fibre consumption from fruits and vegetables improves oxidative stress markers in healthy young adults. Br J Nutr.

[43] McMartin SE, Jacka FN, Colman I (2013). The association between fruit and vegetable consumption and mental health disorders: evidence from five waves of a national survey of Canadians. Prev Med.

[44] Narmaki E, Siassi F, Koohdani F, Qorbani M, Shiraseb F, Ataie-Jafari A (2015). Dietary diversity as a proxy measure of blood antioxidant status in women. Nutrition.

[45] Sedaqat F, Rabiei S, Faria S, Rastmanesh R (2013). Correlates of snacking with stress and depression in obese and non-obese women. J Obes Weight Loss Ther.

[46] Laugero KD, Falcon LM, Tucker KL (2011). Relationship between perceived stress and dietary and activity patterns in older adults participating in the Boston Puerto Rican Health Study. Appetite.

[47] Oliver G, Wardle J (1999). Perceived effects of stress on food choice. Physiol Behav.

[48] Ferguson EL, Gibson RS, Ounpuu S, Sabry JH (1989). The validity of the 24-hour recall for estimating the energy and selected nutrient intake of a group of rural Malawian preschool children. Ecol Food Nutr.

[49] Gibson EL (2006). Emotional influences on food choice: sensory, physiological and psychological pathways. Physiol Behav.

[50] Singh M (2014). Mood, food, and obesity. Front Psychol.

